# Bacterial community analysis of the skin microbiota of cultured Chinese giant salamander infected with *Ranavirus*

**DOI:** 10.3389/fmicb.2024.1356161

**Published:** 2024-04-24

**Authors:** Han Zhang, Hongying Ma, Wei Jiang, Jie Deng, Jianglai Yuan, Cheng Fang, Hu Zhao, Jianlu Zhang, Fei Kong, Hongxing Zhang, Qijun Wang

**Affiliations:** ^1^Shaanxi Key Laboratory of Qinling Ecological Security, Shaanxi Institute of Zoology, Xi’an, China; ^2^Hanzhong Aquatic Development Centre, Hanzhong, China

**Keywords:** bacterial community, microbiome, skin, cultured Chinese giant salamander, Chinese giant salamander iridovirus

## Abstract

Skin microorganisms are an important component of host innate immunity and serve as the first line of defense against pathogenic infections. The relative abundance of bacterial species, microbial community assembly, and secretion of specific bacterial metabolites are closely associated with host health. In this study, we investigated the association between the skin microbiome and *Ranavirus*, and compared the bacterial community assemblage, alpha and beta diversity, and functional predictions of the skin bacterial assemblage in cultured healthy Chinese giant salamanders (*Andrias davidianus*) and individuals infected with Chinese giant salamander iridovirus (GSIV or ADRV). To achieve this, we employed 16S rRNA amplicon sequencing. The results identified Proteobacteria, Firmicutes, Bacteroidota, and Actinobacteriota as the dominant phyla in the diseased and healthy groups. Alpha diversity analysis indicated that the skin bacterial community in the diseased group exhibited no significant differences in bacterial species diversity and lower species richness compared to the healthy group. Beta diversity suggested that the two group bacterial community was quite different. Kyoto encyclopedia of genes and genomes (KEGG) pathway analyze and clusters of orthologous groups of proteins (COG) function predictions revealed that changes and variations occurred in the metabolic pathways and function distribution of skin bacterial communities in two groups.

## 1 Introduction

For amphibians, bacterial communities in the skin microbiota play an important role in the host’s defense mechanism (Longo et al., 2015; Brunetti et al., 2021; Jani et al., 2021). In recent years, the recognition of symbiotic bacterial communities has grown, underscoring their ability to modulate and contribute to host immunity, thereby protecting against multiple host pathogens (Rosenberg et al., 2007; Woodhams et al., 2007; Khosravi and Mazmanian, 2013; Fraune et al., 2015; Bletz et al., 2017; Feng et al., 2022). Notably, investigations have indicated that protection from cutaneous symbiotic bacteria may be linked to specific bacterial metabolites (Becker et al., 2009; Woodhams et al., 2018). For instance, *Janthinobacterium lividum* and *Serratia marcescens* have been shown to produce antifungal and antibacterial metabolites, such as violacein and prodigiosin, respectively, which exert inhibitory effects on factors such as bacteria cell membrane integrity, RNA, or protein synthesis (Woodhams et al., 2018). Improving our understanding of the structure and function of the amphibian skin bacterial communities will help elucidate why some individuals or populations are more (or less) susceptible to infection and disease impacts, thereby mitigating the occurrence of various diseases (Bates et al., 2018).

The Chinese giant salamander (*Andrias davidianus*) is the largest and most ancient extant amphibian species globally and is a critically endangered species in China. In view of the species’ high nutritional, medicinal, and scientific value, the Chinese giant salamander has been artificially farmed in China. Unfortunately, since 2010, the Chinese giant salamander has been confronted with a surge in emerging infectious diseases, which has led to precipitous wild population declines and jeopardized the conservation of Chinese giant salamanders (Dong et al., 2011; Geng et al., 2011; Chen et al., 2018). The spread of disease can be attributed to factors such as high-density intensive farming, amplified international and national trading, and rapid expansion of captive farming operations (Chen et al., 2018; Jiang et al., 2021).

The primary causative agent behind these diseases is the Chinese giant salamander iridovirus (GSIV), also known as *Andrias davidianus ranavirus* (ADRV), which is classified within the *Ranavirus* (RVs) genus of the family Iridoviridae. ADRV is a sizable cytoplasmic double-stranded DNA virus that exhibits rapid transmission, has no control measures, and is associated with a mortality rate surpassing 90% owing to the dearth of effective treatments for affected Chinese giant salamanders (Zhang et al., 2020). *A. davidianus* infected with ADRV often exhibits notable morphological abnormalities, multiple skin hemorrhages, skin ulceration and muscle necrosis of limbs. An anatomical examination always reveals the presence of a significant quantity of yellowish fluid in the abdominal cavity, along with multiple enlarged and discolored viscera and a congested hemorrhagic lung capsule (Jiang et al., 2021). Although significant progress has been achieved in understanding the genomic structure of ADRV, vaccine research and host antiviral immune responses, there is currently no available effective treatment for ADRV (Chen et al., 2018).

To date, the defensive role played by skin bacterial communities in *A. davidianus* infected with ADRV remains relatively unexplored. Given that the microorganisms inhabiting the skin of Chinese giant salamanders have a natural role in defense against pathogens, our study employs state-of-the-art 16S rRNA amplicon sequencing technology to investigate and compare the difference in skin bacterial assemblage between ADRV-infected cultured Chinese giant salamanders and healthy ones. This investigation contributes toward our understanding the association between skin microbiome and ADRV, in addition to helping facilitate the development of effective preventive and therapeutic strategies.

## 2 Materials and methods

### 2.1 Purchase and rearing of experimental animals

In August 2022, six cultured Chinese giant salamanders displaying similar symptoms indicative of ADRV infection were acquired from Hanzhong Longtoushan Aquaculture Co., Ltd. The salamanders were housed in containers with regular water changes every three days and were maintained at room temperature (20 ± 2°C) within a laboratory setting. An additional six healthy cultured Chinese giant salamanders were procured from the same company for the control group and provided identical environmental and temperature conditions. All Chinese giant salamanders were of the same age (two years old), and sex (males), belonged to the same litter, had a similar diet (freshwater fish purchased in fixed markets), and were kept in similar tanks (culture pond at the farm and 0.5 m × 1 m tanks in our laboratory). Sex was confirmed using the paraffin sections of the gonads.

### 2.2 Disease diagnosis

First, morphological observations of the cultured Chinese giant salamanders were made using anatomical methods. Subsequent molecular biology tests were conducted to further confirm whether the cause was ADRV.

DNA was extracted from the liver tissue of cultured Chinese giant salamanders from the diseased and healthy groups using a viral genomic DNA/RNA extraction kit (catalog# DP315, Beijing Tiangen Biotech Co., Ltd.). All of the liver tissue were separately preserved in a refrigerator at −80°C to avoid possible *Ranavirus* contamination. The extracted DNA was then subjected to detection through polymerase chain reaction (PCR) utilizing MCP1 primer pairs that were designed within our laboratory.

The PCR reaction system consisted of 2 × Taq PCR Mix II (catalog# KT211-02, Tiangen Biotech) in a volume of 10 μL, with 1 μL each of upstream and downstream primers (10 μM), 1 μL template DNA, and 7 μL deionized water (ddH_2_O), resulting in a total reaction volume of 20 μL. The reaction procedure involved an initial pre-denaturation step at 95°C for 10 min, followed by denaturation at 95°C for 30 s, annealing at 58°C for 30 s, extension at 72°C for 35 cycles, and a final extension step at 72°C for 10 min. The length of the resulting target gene fragment was 456 bp.

### 2.3 Skin bacterial microbial sample collection

Each individual (in both the experimental and control groups) underwent one time for data collection and analysis. The following method was employed to collect bacterial communities from the skin of the cultured Chinese giant salamanders: first, the salamanders were handled using sterile high-density polyethylene gloves, placed into a clean water tank, and rinsed thrice with 1 L sterile water to eliminate transient bacteria from the skin surface. Subsequently, the skin on the back, abdomen, and limbs was swabbed 30 times using a sterile skin swab. The swab was placed in 2 mL of DNA storage solution and temporarily stored at 4 °C. Finally, the collected samples were preserved into an ice box with dry ice and transported to Beijing Biomarker Technologies Co. Ltd. for subsequent sequencing analysis.

### 2.4 16S rRNA sequencing and statistical analyses

Total genomic DNA was extracted from the skin samples of cultured Chinese giant salamanders using the TGuide S96 Magnetic Soil/Stool DNA Kit (Tiangen Biotech) following the manufacturer’s instructions. The hypervariable regions V4-V5 of the bacterial 16S rRNA genes were amplified using the primer pairs 515F: 5′-GTGYCAGCMGCCGCGGTAA-3′ and 907R: 5′-CCG YCAATTYMTTTRAGTTT-3′ (Parada et al., 2016). The resulting PCR products were analyzed on an agarose gel and subsequently purified using the Omega DNA purification kit (Omega Inc., Norcross, GA, USA). The purified PCR products were collected and subjected to paired-end sequencing (2 × 250 bp) on the Novaseq 6000 platform (Illumina Inc., San Diego, CA, USA).

The bioinformatics analysis of this study was performed with the aid of the BMKCloud.^1^ According to single nucleotide quality, raw data was primarily filtered by Trimmomatic (version 0.33) (Bolger et al., 2014). Identification and removal of primer sequences was processed by Cutadapt (version 1.9.1) (Martin, 2011). Paired-end reads obtained from previous steps were assembled by USEARCH (version 10), followed by chimera removal using UCHIME (version 8.1) (Edgar et al., 2011; Edgar, 2013). Subsequently, clean reads were conducted on feature classification to output a ASVs (amplicon sequence variants) by dada2 (Callahan et al., 2016). Taxonomy annotation of the ASVs was performed based on the Naive Bayes classifier in QIIME2 using the SILVA database (release 138.1) with a confidence threshold of 70% (Quast et al., 2013). The Alpha diversity was calculated and displayed by the QIIME2 and ggplot2 software, respectively. Beta diversity was determined to evaluate the degree of similarity of microbial communities from different samples using QIIME2 (Bolyen et al., 2019). Principal co-ordinates analyses (PCoA, based on dissimilarity matrices) and PERMANOVA (Adonis function in the Vegan package) were employed to visualize the dissimilarity of beta diversity (Dixon, 2003; Anderson, 2017). Furthermore, we employed linear discriminant analysis (LDA) effect size (LEfSe) to test for significant taxonomic differences among group (Segata et al., 2011). A logarithmic LDA score of 4.0 was set as the threshold for discriminative features. Functional predictions were conducted using the PICRUSt2 software (Douglas et al., 2020).

## 3 Results

### 3.1 Disease diagnosis

**FIGURE 1:**
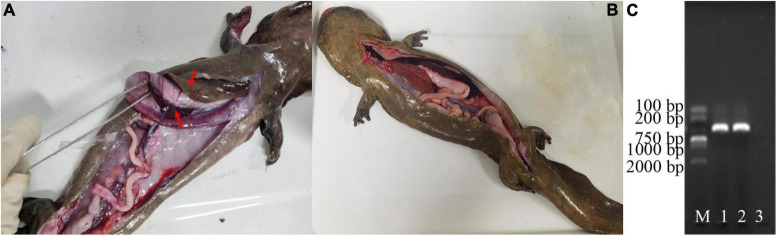
Diagnosis of diseases of the Chinese giant salamanders. Anatomical features of a diseased **(A)**, and a healthy **(B)** Chinese giant salamander. PCR results of the DNA samples extracted from the liver tissue of them **(C)**. The gel image shows the bands corresponding to the amplified DNA fragments. M represents the molecular weight marker, 1–2 denotes the DNA samples from the two of them diseased Chinese giant salamanders, and 3 indicate the blank control. The results showed that the diseased Chinese giant salamander was infected with ADRV.

Notable behavioral and physiological differences were observed between the healthy and diseased groups (Figures 1A, B). Specifically, diseased individuals exhibited clinical symptoms such as sluggish behavior, reduced appetite, occasional vomiting, and increased mucus secretion from the body surface. Bleeding was observed in the lower jaw and on various locations of the limbs. Furthermore, the limbs displayed signs of skin ulceration and muscle necrosis, and one individual lost a forelimb. An anatomical examination revealed clinical signs in the six diseased Chinese giant salamanders that are consistent with the classic clinical signs of disease caused by ADRV infection, including: a significant quantity of yellowish fluid in the abdominal cavity, enlarged grayish-white liver, enlarged purple-black spleen, enlarged hemorrhagic kidneys, and congested hemorrhagic lung capsule (Figure 1A). The PCR results of the DNA samples extracted from the liver tissue of diseased Chinese giant salamanders unequivocally confirmed their infection with ADRV (Figure 1C), while the other six were uninfected. Therefore, it can be conclusively stated that the disease observed in the six Chinese giant salamanders was attributable to ADRV infection.

### 3.2 Sample sequencing and sampling depth validation

Using sequencing, data filtering, and sequence splicing techniques, a total of 502,639 sequences were obtained from the group labeled as “A” (A1-A6), which comprised samples from six Chinese giant salamanders infected with ADRV. Similarly, 449,115 sequences were obtained from the group labeled as “B” (B1-B6), consisting of samples from six healthy Chinese giant salamanders (Table 1). These sequences were further processed and clustered into amplicon sequence variants (ASVs). Venn diagram analysis revealed 5064 ASVs in the diseased group, 736 ASVs in the healthy group, 147 ASVs common to both groups (Supplementary Figure 1 in the Supplementary material).

**TABLE 1:** Sequencing results and amplicon sequence variants (ASVs) in diseased and healthy groups.

Sample	Sequence number	ASV number	Abundance index		Diversity index	
			Chao1	ACE	Shannon	Simpson
A1	70439	380	380	380	3.681	0.6567
A2	112830	755	755	755	5.0157	0.7571
A3	81756	1096	1,096	1,096	7.0837	0.9213
A4	88880	1846	1,846	1,846.1472	6.8919	0.9223
A5	76938	968	968	968	6.7999	0.9071
A6	71796	840	840	840	6.2483	0.8949
B1	69972	126	126	126	3.5015	0.8378
B2	73069	227	227	227	5.6605	0.943
B3	75318	166	166	166	4.5877	0.8436
B4	77102	184	184	184	5.3631	0.8921
B5	77707	109	109	109	4.0535	0.8065
B6	75947	159	159	159	4.5408	0.8374

All 12 sample dilution curves exhibited a flat pattern, indicating an adequate sequence depth. The rarefaction curves of detected bacterial species in the skin microbiota reached saturation with the increasing number of samples, which suggests the skin microbiota in our population captured most bacterial members (Supplementary Figure 2A in the Supplementary material). The relative abundance curves of the 12 samples displayed substantial fluctuations across the horizontal axis, signifying considerable variation in bacterial relative abundance among the samples (Supplementary Figure 2B).

### 3.3 Alpha diversity

**FIGURE 2:**
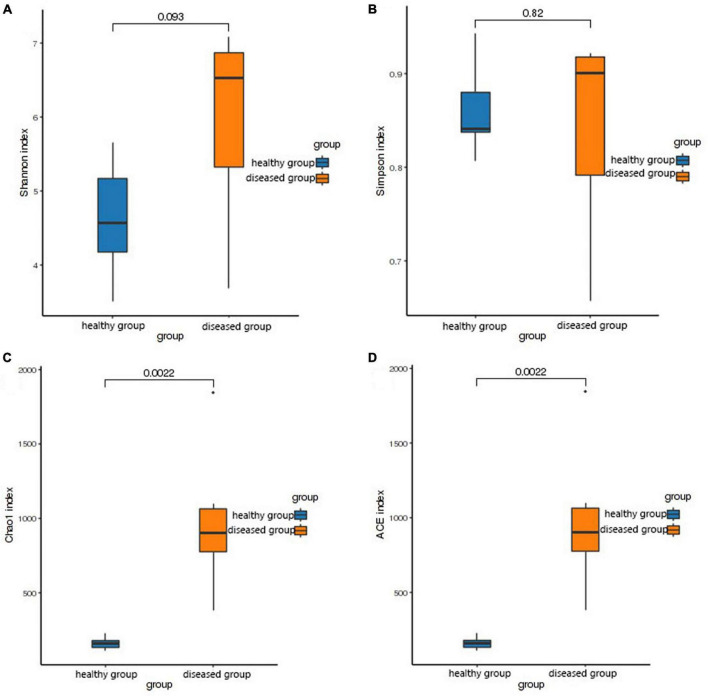
Box plot of differences between groups of alpha diversity indices. The horizontal coordinates are the group names, and the vertical coordinates are the values of the corresponding alpha diversity indices. **(A)** Wilcoxon test of Shannon index, **(B)** Wilcoxon test of Simpson index, **(C)** Wilcoxon test of Chao1 index, **(D)** Wilcoxon test of ACE index.

The Shannon and Simpson (species diversity) did not differ significantly between the two groups (*P* > 0.05, Figures 2A, B). However, the Chao1 and ACE indices (species richness) revealed significantly higher skin bacteria species richness in the healthy group than that in the diseased group (*P* < 0.01, Figures 2C, D). This finding was further supported by the rank abundance curves.

### 3.4 Comparison of bacterial community structure

**FIGURE 3:**
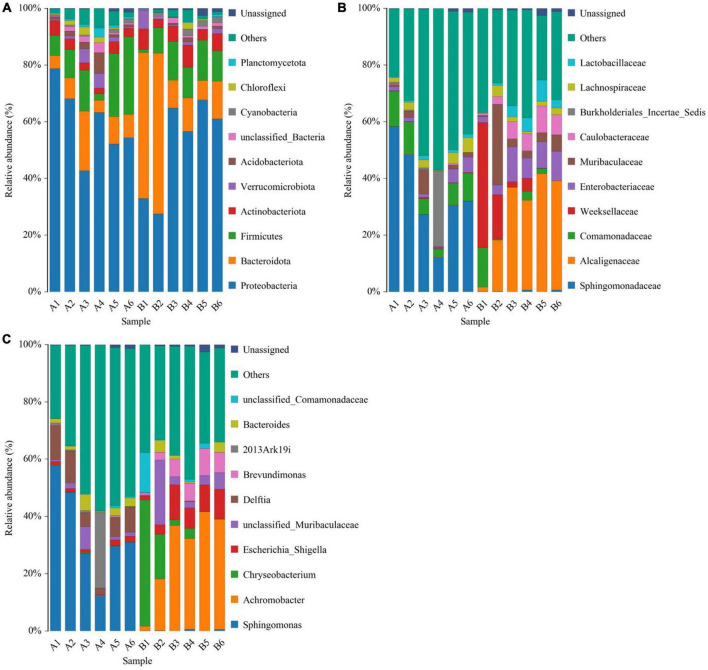
Histogram of species distribution at different levels of diseased group (A1-A6) and healthy group (B1-B6). **(A)** Histogram of species distribution at the phylum level. **(B)** Histogram of species distribution at the family level. **(C)** Histogram of species distribution at the genus level. Horizontal coordinates are sample names; vertical coordinates are relative abundance percentages. Different colors indicate different species; stacked columns are the top 10 taxa in relative abundance at each taxonomic level.

The top five phyla observed in the diseased group (A1-A6) were Proteobacteria (59.9 ± 5.23%), Firmicutes (13.9 ± 3.86%), Bacteroidota (9.18 ± 2.51%), Actinobacteriota (3.46 ± 0.477%), and Acidobacteriota (2.31 ± 1.04%). In healthy group (B1-B6), the top five phyla (in terms of average relative abundance) were Proteobacteria (51.8 ± 7.02%), Bacteroidota (25 ± 9.27%), Firmicutes (9.93 ± 1.93%), Actinobacteriota (5.52 ± 0.79%), and Verrucomicrobiota (1.77 ± 0.996%) (Figure 3A).

The top five families in the diseased group were Sphingomonadaceae (34.8 ± 6.66%), Comamonadaceae (8.25 ± 1.51%), Burkholderiales_Incertae_Sedis (4.73 ± 4.36%), Lachnospiraceae (2.74 ± 0.695%) and Muribaculaceae (2.41 ± 1.25%). The top five families in healthy group in terms of average relative abundance were Alcaligenaceae (28.1 ± 0.28%), Weeksellaceae (11.1 ± 7.04%), Enterobacteriaceae (7.45 ± 1.64%), Muribaculaceae (7.2 ± 4.31%), and Caulobacteraceae (5.38 ± 1.28%) (Figure 3B).

For microbial genera, the top five observed in diseased group (A1-A6) based on average relative abundance were *Sphingomonas* (34.4 ± 6.65%), *Delftia* (7.63 ± 1.54%), *2013Ark19i* (4.73 ± 4.36%), *Bacteroides* (2.39 ± 0.777%), and unclassified_*Muribaculaceae* (2.25 ± 1.18%). In healthy group (B1-B6) group, the top five families in terms of mean relative abundance were *Achromobacter* (28.1 ± 6.28%), *Chryseobacterium* (10.8 ± 7.06%), *Escherichia*_*Shigella* (7.31 ± 1.72%), unclassified_*Muribaculaceae* (6.15 ± 3.36%), and *Brevundimonas* (5.38 ± 1.28%) (Figure 3C).

### 3.5 Beta diversity

**FIGURE 4:**
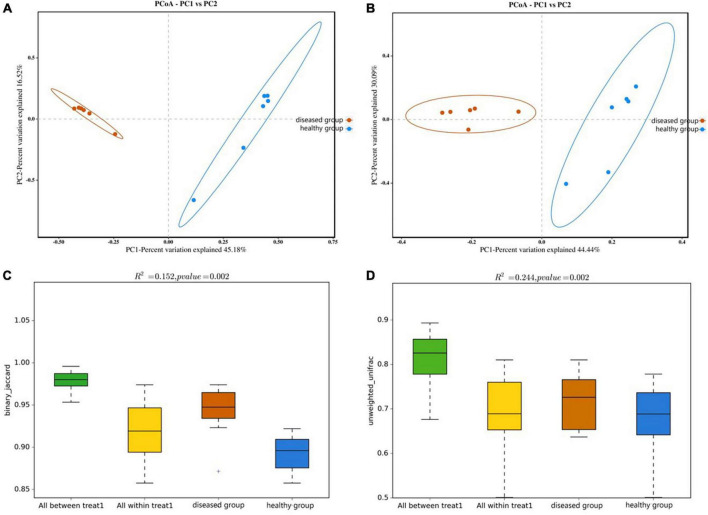
PCoA analyze **(A,B)** and PERMANOVA analysis **(C,D)** of the beta diversity of the skin bacterial community. **(A,C)** Binary jaccard distance; **(B,D)** unweighted UniFrac distance. Each point represents the skin bacterial community of an individual sample in PCoA analyze graph. In PERMANOVA analysis graph, Vertical sits indicate the Beta distance; the box plots above “All between *” represent the Beta distance data for all samples between groups, the box plots above “All within” represent the Beta distance data for all samples within groups, and the later box plots are Beta distance data between samples within groups of different subgroups, respectively.

PCoA analysis (the Bray-Curtis algorithm and the Weighted- Unifrac algorithm) revealed that the samples of two groups were far apart in the direction of the PC1 axis (Figures 4A, B), which meant that there was a large gap in their bacterial community, and the PC1 axis was the main factor contributing to this difference. PERMANOVA analysis (*p* = 0.002, Figures 4C, D) verified that there were significant differences in bacterial community between the diseased and healthy groups.

### 3.6 Analysis of biomarker in different groups

**FIGURE 5:**
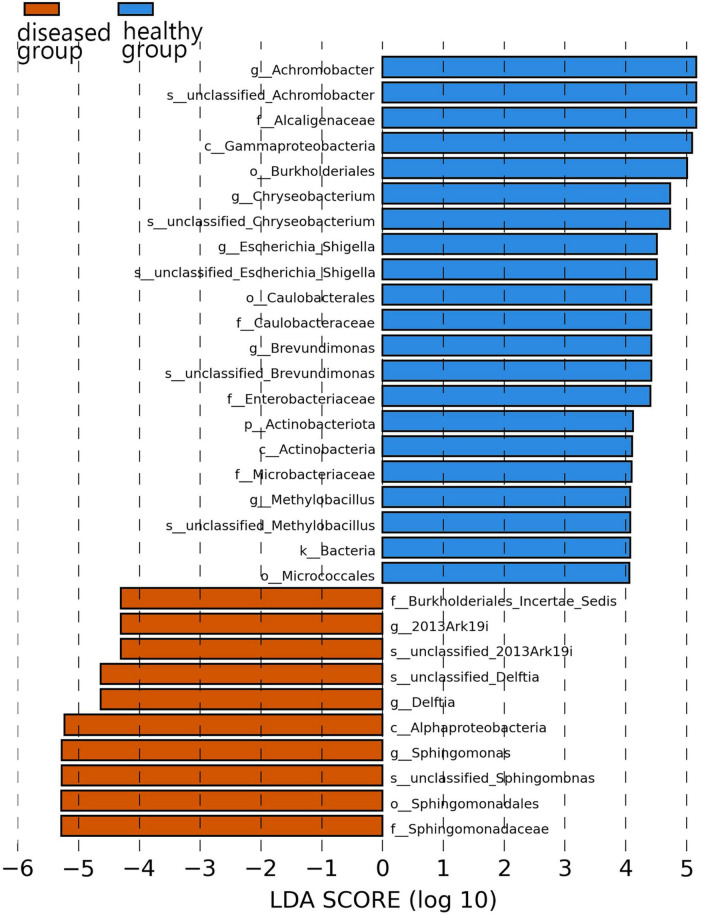
Histogram of the distribution of LDA values. The vertical axis represents the taxonomic units exhibiting significant differences between the groups, while the horizontal axis displays bar graphs illustrating the logarithmic scores of LDA analysis for each respective taxonomic unit.

Specific bacterial populations with differential relative abundances between the two sample groups were analyzed using LEfSe (Figure 5). The results revealed significant differences at the genus level. In the diseased group, three genera—namely, *Sphingomonas*, *Delftia*, and *2013Ark19i* were biomarkers of statistically significant differences; *2013Ark19i* was found exclusively in the diseased group. In contrast, the healthy group exhibited the presence of five genera: *Chryseobacterium*, *Brevundimonas*, *Achromobacter*, *Methylobacillus*, and *Escherichia*_*Shigella*. The abundance of *Methylobacillus* and *Escherichia*_*Shigella* were the most abundant of the five genera, and were exclusive to the healthy group.

### 3.7 Functional prediction of the bacterial community

**FIGURE 6:**
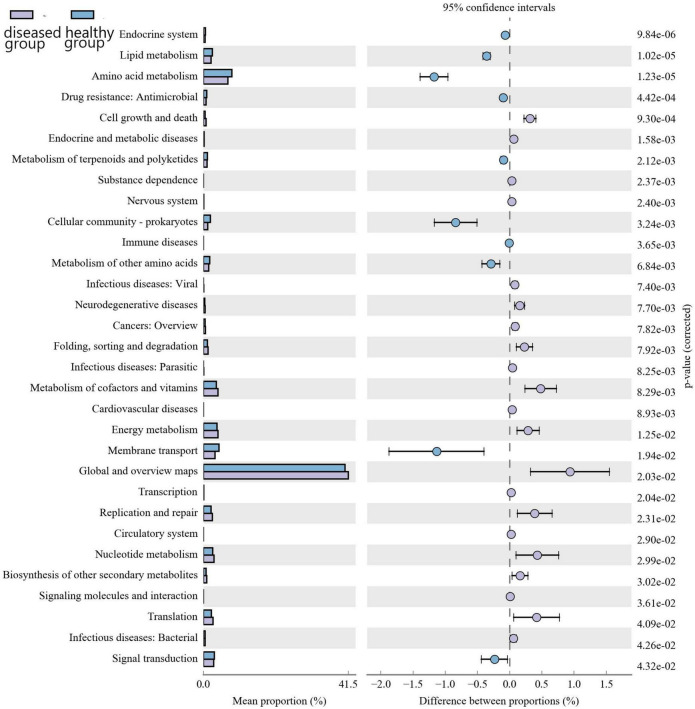
Differential analysis of the KEGG metabolic pathway at the second level. The proportion of relative abundance of different functions in two groups of samples is shown on the left side of the graph, the proportion of difference in the relative abundance of functions in the 95% confidence interval is shown in the middle, and the rightmost value is the *p*-value.

Kyoto encyclopedia of genes and genomes (KEGG) pathway prediction analysis of the gene functions of the skin microbiota present in cultured Chinese giant salamanders revealed their involvement in various functions, including global and overview maps, amino acid metabolism, energy metabolism, membrane transport, and metabolism of cofactors and vitamins (Figure 6). However, functional gene relative abundances in skin bacterial communities differed significantly between the diseased and healthy groups, indicating that changes and variations in the metabolic pathways of skin bacterial communities occurred in two groups.

**FIGURE 7:**
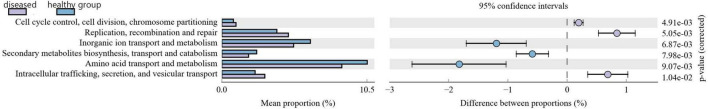
Statistical graph of COG functional classification. The proportion of relative abundance of different functions in two groups of samples is shown on the left of the figure, the proportion of difference in the relative abundance of functions in the 95% confidence interval is shown in the middle, and the rightmost value is the *p*-value.

Clusters of orthologous groups of proteins (COG) functional prediction analysis demonstrated that the relative abundance of functional genes related to cell cycle control, cell division, chromosome partitioning, replication, recombination and repair, intracellular transport, secretion, and vesicular transport was higher in the diseased Chinese giant salamander group than that in the healthy group (Figure 7). These findings suggest that the aforementioned functions may contribute to the defense against ADRV and provide valuable insights for further research concerning ADRV in Chinese giant salamanders.

## 4 Discussion

In this study, skin swabs were collected from cultured Chinese giant salamanders to investigate differences in skin bacterial communities between diseased and healthy individuals using high-throughput 16S rRNA gene sequencing. The results show that ADRV could cause changes in the skin bacterial community of cultured Chinese giant salamander, which including community structure, alpha and beta diversity, and functional aspects.

At the phylum level, Proteobacteria, Firmicutes, Bacteroidetes, and Actinobacteria were found to be dominant in both sets of samples, which is consistent with other research on skin bacteria in anuran amphibians (Walke et al., 2014; Jiménez et al., 2019; Yang et al., 2020). These findings indicate that amphibian skin can serve as a colonization site for specific bacteria, and the presence of ADRV infection does not disrupt the colonization of the bacteria of Proteobacteria, Firmicutes, Bacteroidetes, and Actinobacteria. However, significant changes were observed in the relative abundances of these bacteria. Proteobacteria and Firmicutes showed increased relative abundances in cultured Chinese giant salamanders infected with ADRV compared with those of healthy individuals, and the relative abundances of Bacteroidetes and Actinobacteria were lower in the diseased group. This seems to indicate that apart from Proteobacteria (Woodhams et al., 2007; Zhu et al., 2022), Firmicutes is also related to amphibian defense and immunity. Additionally, Acidobacteria showed an increased relative abundance and ranked fifth in the diseased group, replacing Verrucomicrobiota, which ranked fifth in the healthy group and ranked down to sixth in diseased group. The phylum Acidobacteria is widespread and prefers an acidic environment (Kalam et al., 2020), which indicates that the skin PH of Chinese giant salamanders may have changed when they infected with ADRV.

At the family and genus levels, only Muribaculaceae and unclassified_Muribaculaceae within this family were dominant in both sample groups. Muribaculaceae is widespread in animal guts and versatile with respect to complex carbohydrate degradation (Lagkouvardos et al., 2019). Muribaculaceae is also found in the skin of cultured Chinese giant salamander, indicating that Muribaculaceae has a broader range beyond the animal gut. Notably, the highest reported abundance of Enterobacteriaceae or Pseudomonadaceae in anuran amphibians differed from that of the cultured Chinese giant salamander in this study (Rebollar et al., 2018; Yang et al., 2020), and could be due to host species differences. In the diseased Chinese giant salamander group, Sphingomonadaceae, along with the genus *Sphingomonas* belonging to this family, exhibited the highest relative abundance. *Sphingomonas* possesses a unique ability to degrade polycyclic aromatic hydrocarbons (PAHs), which are known for their toxic, genotoxic, mutagenic and/or carcinogenic properties (Zhou et al., 2016). The high relative abundance of *Sphingomonas* indicates that there is potential contamination of PAHs in the environment of Chinese giant salamanders infected with ADRV compared to the healthy group. However, whether PAHs are directly associated with ADRV is unclear. Although from Figure 5 it appears that the genus 2013Ark19i of diseased group exhibit high abundance, the relative abundance of 2013Ark19i is higher only in sample A4 (26.5%) but very lower in others (Figure 3C); therefore the relative abundance 26.5% is considered as an outlier. However, what cannot be ignored is that the genus 2013Ark19i was exclusively present in the diseased group, further investigations are necessary to determine whether this genus is associated with ADRV because there are few descriptions about this genus. Therefore, identifying microbial signatures linked to the variation in disease susceptibility might provide guidance for the treatment of diseases and the development of bacterial applications.

Our analysis of the alpha and beta diversity index results suggests that the richness of the related bacterial composition decreased in diseased group and that the structure of the bacterial community differs between the two groups. Thus, ADRV infection likely alters the structure of bacterial communities, thereby sabotaging the protective effect of these symbiotic bacteria.

The functional prediction analyses demonstrated that some functional genes may play a more important role in defense against viruses; however, whether and how they function will be addressed in our future studies. Notably, the prediction of community function by 16S rRNA gene sequencing has limitations. On the one hand, a high degree of similarity in 16S rRNA sequences across species does not mean that shared functional genes are highly similar (Sevigny et al., 2019). On the other hand, the presence of a particular microorganism does not mean that the microorganism exerts its relevant biological function. Although the prediction method cannot replace macro-genome sequencing experiments, the method remains important for guiding the design of subsequent macro-genome experiments.

The skin microbial community plays an important role in the host’s defense mechanisms (Brunetti et al., 2021). When ADRV infects the Chinese giant salamander, the skin microecology and other immune organs change accordingly (Jiang et al., 2021). In this study, the infected individuals exhibited the disease at varying degrees at the tissue or organ levels. However, whether the dysregulation of the skin bacterial community in the cultured Chinese giant salamanders was caused by the tissue lesions and whether ADRV directly acted on the skin to cause changes in the skin bacterial community remains poorly understood. Therefore, these topics require further research. What can be concluded, however, is that changes in the skin symbiotic bacterial community are related to ADRV infection.

In addition, mixed bacterial-viral infections may occur in Chinese giant salamanders infected with ADRV, along with secondary bacterial infections caused by pathogens such as *Aeromonas hydrophila* and *Aeromonas veronii* (Zhang et al., 2020). The two bacterial infections exhibit similarities to those observed in cultured Chinese giant salamanders infected with ADRV. In this study, a small number of *A. veronii* bacteria were detected within the skin bacterial community of one diseased Chinese giant salamander. Thus, this particular salamander may have experienced a mixed infection involving both viruses and bacteria. One possible explanation for secondary bacterial infections in the Chinese giant salamander may be that ADRV infection reduces the diversity of its symbiotic microbiome, which could increase susceptibility to bacteria (Becker and Harris, 2010; Feng et al., 2022). The Chinese giant salamander can be infected by ADRV regardless of developmental stage. ADRV-caused mass die-offs can occur whenever environmental triggers form suitable conditions for an outbreak. Thus, implementing effective early prevention and improving the quality of daily protection against ADRV in the Chinese giant salamander is essential. Notably, owing to early prevention and timely control, ADRV at Hanzhong Longtoushan Farm last year did not spread on a large scale, and only six Chinese giant salamanders were found to be infected with ADRV. To improve the understanding of Chinese giant salamander skin microbial ecology, a larger number of diseased and healthy Chinese giant salamanders are required.

## 5 Conclusion

In this investigation, Proteobacteria, Firmicutes, Bacteroidetes, and Actinobacteria were identified as the dominant phyla in samples from the diseased and healthy groups; however, significant differences were observed in the relative abundances of these bacteria between groups. Alpha diversity analysis indicated no significant difference in species diversity, but the diseased group exhibited lower species richness than the healthy group. Additionally, the skin bacterial communities of cultured Chinese giant salamanders exhibited significantly different beta diversities and may have undergone differential metabolic pathways and functional changes after *Ranavirus* infection. Our findings provide a novel insight into the skin bacterial community of Chinese giant salamanders; however, understanding how the skin microbiota interact with *Ranavirus* requires further research.

## Data availability statement

The datasets presented in this study can be found in online repositories. The names of the repository/repositories and accession number(s) can be found below: https://www.ncbi.nlm.nih.gov/, NCBI BioProject, PRJNA1034501.

## Ethics statement

The animal studies were approved by the Animal Ethics Committee of the Shaanxi Institute of Zoology. The studies were conducted in accordance with the local legislation and institutional requirements. Written informed consent was obtained from the owners for the participation of their animals in this study.

## Author contributions

HanZ: Conceptualization, Writing – original draft. HM: Writing – review & editing. WJ: Conceptualization, Writing – review & editing. JD: Conceptualization, Writing – review & editing. JY: Methodology, Writing – review & editing. CF: Data curation, Writing – review & editing. HuZ: Supervision, Writing – review & editing. JZ: Supervision, Writing – review & editing. FK: Supervision, Writing – review & editing. HonZ: Methodology, Writing – review & editing. QW: Methodology, Writing – review & editing.

## Footnotes

1
http://www.biocloud.net/
