# Supplementary material for: Bacterial community analysis of the skin microbiota of cultured Chinese giant salamander infected with Ranavirus
Source: Front Microbiol. 2024 Apr 24;15:1356161. doi: 10.3389/fmicb.2024.1356161 (PMC11076746; doi:10.3389/fmicb.2024.1356161)
Supplement: Supplementary file 1 [file Table_1.DOCX]

**Supplementary Figures**


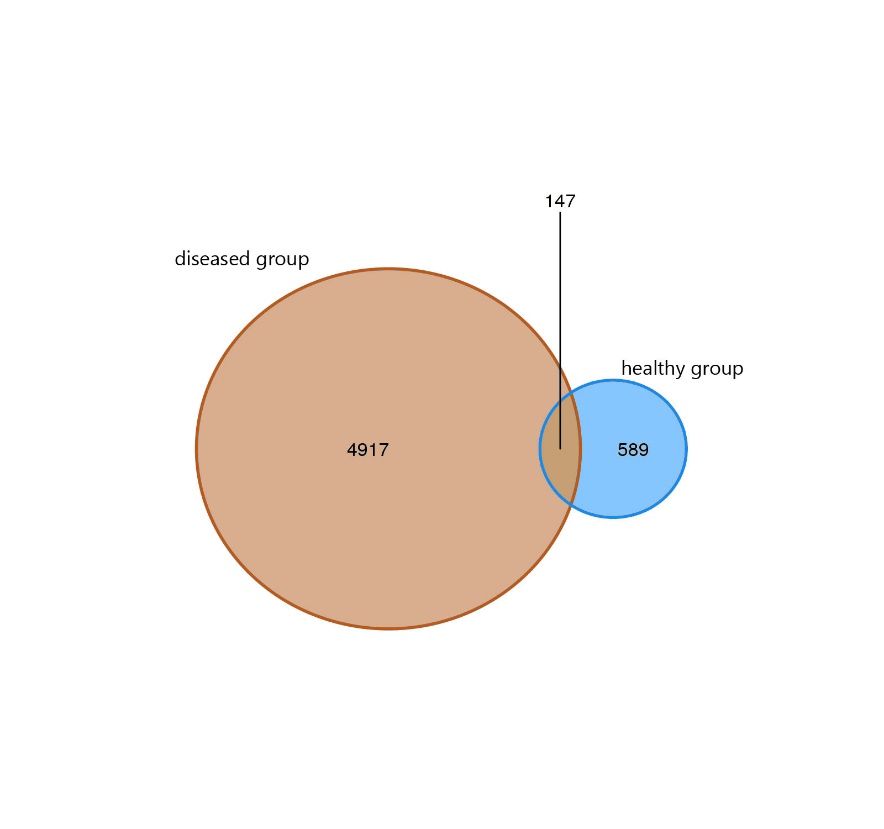


**Supplementary Figure 1** Venn diagram presenting number of shared, uniqueASVs between two groups.


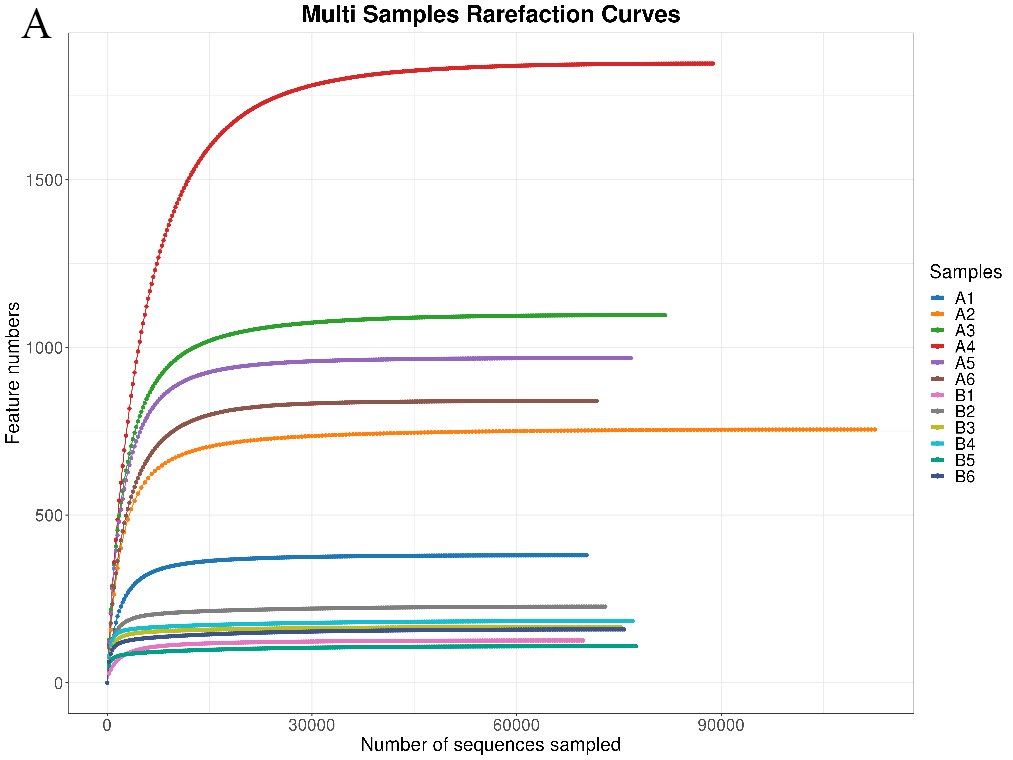

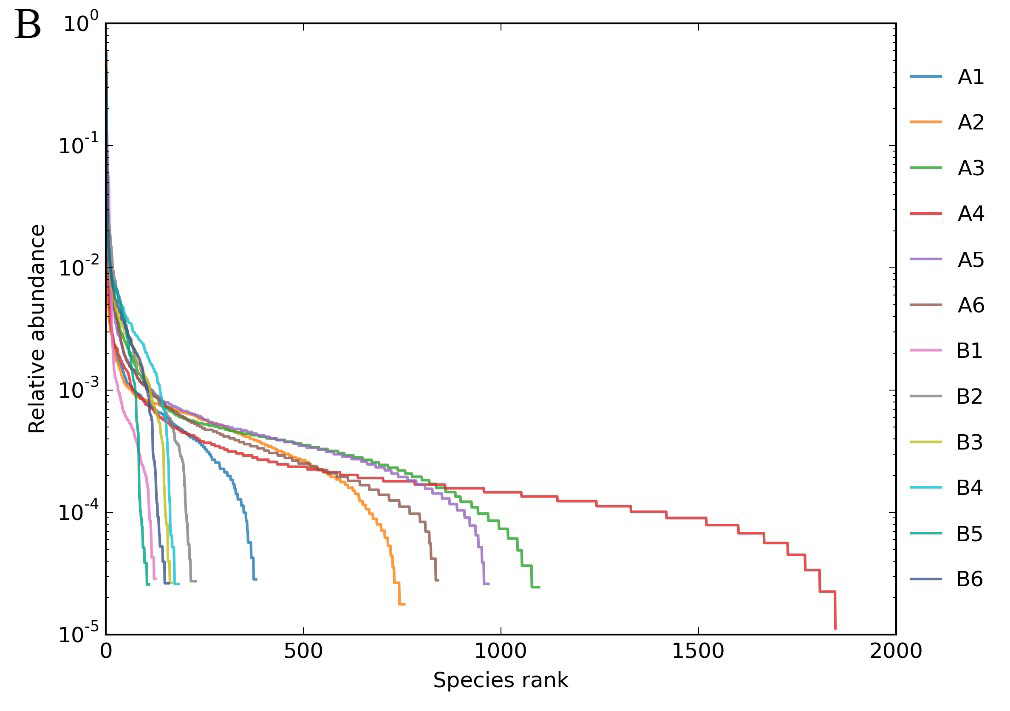


**Supplementary Figure 2** Sample rarefaction curves and rank abundance curves. A) Sample rarefaction curves. The horizontal coordinate is the number of randomly selected sequencing strips, the vertical coordinate is the number of Features obtained based on the number of sequencing strips, each curve represents one sample. B) Sample rank abundance curves. The horizontal coordinate is the ordinal number sorted by the abundance of ASVs, the vertical coordinate is the relative abundance of the corresponding ASVs.
